# Impaired Right and Left Ventricular Longitudinal Function in Patients with Fibrotic Interstitial Lung Diseases

**DOI:** 10.3390/jcm9020587

**Published:** 2020-02-21

**Authors:** Agostino Buonauro, Ciro Santoro, Maurizio Galderisi, Angelo Canora, Regina Sorrentino, Roberta Esposito, Maria Lembo, Mario Enrico Canonico, Federica Ilardi, Valeria Fazio, Bruno Golia, Alessandro Sanduzzi Zamparelli, Maria Luisa Bocchino

**Affiliations:** 1 Department of Advanced Biomedical Sciences, Federico II University Hospital, 80131 Naples, Italy; stino84@gmail.com (A.B.); cirohsantoro@gmail.com (C.S.); rejinasorrentino@gmail.com (R.S.); robyeire@tin.it (R.E.); mari.lembo@gmail.com (M.L.); mecanonico@me.com (M.E.C.); fedeilardi@gmail.com (F.I.); 2 Department of Clinical Medicine and Surgery, Federico II University, Monaldi Hospital, 80131 Naples, Italy; a.canora@hotmail.it (A.C.); sanduzzi@unina.it (A.S.Z.); marialuisa.bocchino@unina.it (M.L.B.); 3 Mediterranea Cardiocentro, 80122 Naples, Italy; valeria.fazio1986@gmail.com (V.F.); drbrunogolia@gmail.com (B.G.)

**Keywords:** Lung diseases, Echocardiography, Pulmonary hypertension, Heart failure, Diagnosis

## Abstract

**Background:** Left ventricular (LV) and right ventricular (RV) dysfunction is recognized in idiopathic pulmonary fibrosis (IPF). Little is known about cardiac involvement in non-idiopathic pulmonary fibrosis (no-IPF). This issue can be explored by advanced echocardiography. **Methods:** Thirty-three clinically stable and therapy-naive fibrotic IPF and 28 no-IPF patients, and 30 healthy controls were enrolled. Exclusion criteria were autoimmune systemic diseases, coronary disease, heart failure, primary cardiomyopathies, chronic obstructive lung diseases, pulmonary embolism, primary pulmonary hypertension. Lung damage was evaluated by diffusion capacity for carbon monoxide (DLCO_sb_). All participants underwent an echo-Doppler exam including 2D global longitudinal strain (GLS) of both ventricles and 3D echocardiographic RV ejection fraction (RVEF). **Results:** We observed LV diastolic dysfunction in IPF and no-IPF, and LV GLS but not LV EF reduction only in IPF. RV diastolic and RV GLS abnormalities were observed in IPF versus both controls and no-IPF. RV EF did not differ significantly between IPF and no-IPF. DLCO_sb_ and RV GLS were associated in the pooled pulmonary fibrosis population and in the IPF subgroup (β = 0.708, *p* < 0.001), independently of confounders including pulmonary arterial systolic pressure. **Conclusion:** Our data highlight the unique diagnostic capabilities of GLS in distinguishing early cardiac damage of IPF from no-IPF patients.

## 1. Introduction

Interstitial lung diseases (ILDs) include more than 200 disorders, characterized by a variable degree of inflammation and fibrosis leading to an often irreversible loss of lung function, wide spectrum in the clinical course, treatment, and prognosis. ILDs can be broadly divided in those without any identifiable cause, that is, idiopathic interstitial pneumonias, and those with identifiable factors such as environmental/occupational exposure, infections, autoimmune systemic diseases, drugs, and radiations [1]. Among idiopathic interstitial pneumonia, idiopathic pulmonary fibrosis (IPF) is the most common form, affecting 30 persons per 100,000 in the general population, and as many as 175 persons per 100,000 in the age group of >75 years [2]. Despite recent advances in pharmacotherapy [3], IPF is a poor-prognosis disease, with a rapidly progressive and debilitating clinical progression [4,5,6,7].

Because of its worse prognosis and challenging treatment, pneumological and cardiac aspects of IPF have been deeply investigated. Respiratory function declines along with disease progression, and changes in lung diffusion capacity of carbon monoxide (DLCO) and forced vital capacity (FVC) are both independent predictors of worse prognosis [1]. Pulmonary arterial hypertension (PAH) is frequently found in the early stages of IPF and the outcome is directly related to the capacity of right ventricular (RV) function to adapt to the elevated afterload [8,9]. The combination of severe vascular and fibrotic abnormalities induces changes in right ventricular (RV) structure and function until heart failure onset [1]. RV enlargement and dysfunction, as evaluated by standard echocardiography, have been well described in IPF and can be used to identify patients with high risk of mortality [10]. Also, an impairment of left ventricular (LV) diastolic filling has been observed, whereas LV systolic function appears to be preserved [11].

Speckle Tracking Echocardiography (STE) has shown to be suitable for diagnosing early cardiac dysfunction in IPF patients [12,13]. Conversely, LV and RV function have been poorly explored by advanced echo technologies in fibrotic ILDs other than IPF (defined in this study as no-IPF) and no comparison of strain and 3D echocardiographic imaging exists between IPF and no-IPF patients. Accordingly, the aim of the present study was to analyze LV and RV structure and function in patients with ILDs, including both IPF and no-IPF, by using 2D strain to both ventricles and 3D to the right ventricle. Relationships between lung function and cardiac parameters were also evaluated. In relation with the relative rare rate of IPF, this was an exploratory, not confirmatory, study and sample size and power calculations could not be performed.

## 2. Experimental Section

### 2.1. Study Population

The study population included 61 patients with clinically stable and therapy-naive fibrotic ILDs, referred at the time of first diagnosis to the Respiratory Diseases Division of the Federico II University, Monaldi Hospital (Naples, Italy), between October 2016 and October 2018. Thirty-three patients were affected by IPF (IPF group) and 28 had fibrotic ILDs other than IPF (no-IPF group), including idiopathic nonspecific interstitial pneumonia (*n* = 14), chronic hypersensitivity pneumonia (*n* = 9), and smoking-related desquamative interstitial pneumonia (*n* = 5). Diagnosis of IPF was made according to the 2011 international diagnostic criteria [2]. Exclusion criteria were autoimmune systemic diseases, coronary artery disease and previous myocardial infarction, more than mild valvular heart disease, overt heart failure, primary cardiomyopathies, atrial fibrillation, congenital heart disease, and inadequate imaging quality. Patients with acute exacerbation of the underlying ILDs and those with concomitant chronic obstructive lung disease were excluded as well. Thirty healthy subjects matched for age and sex, referred for voluntary cardiovascular screening to the Interdepartmental Laboratory of Echocardiography of the Federico II University (Naples, Italy), entered the study as the control group.

The study was conducted in accordance with the amended Declaration of Helsinki and approved by the Institutional Ethical Committee (Protocol 1129, 4 August 2015). All patients gave their written informed consent at enrollment. Patient data were collected in an anonymous way.

### 2.2. Lung Function Evaluation

Spirometry, lung volume measurement, and determination of the haemoglobin-adjusted single-breath diffusing lung capacity of carbon monoxide (DLCO_sb_) were performed using a computer-assisted spirometer (Quark PFT 2008 Suite Version Cosmed Ltd, Rome, Italy) according to international standards [14,15,16]. Arterial gas analysis was performed at rest while patients were breathing ambient air. The six-minute walk test (6-MWT) was performed by trained hospital staff according to international guidelines [17].

### 2.3. Echocardiographic Examination

Standard echo-Doppler exam, including STE of both ventricles and 3D echocardiography of the right ventricle, was performed by a Vivid E9 ultrasound machine (GE Healthcare, Horten, Norway), using a 2.5 MHz transducer with harmonic capability, according to the standards of our laboratory [18,19,20] and the echo report standardization of the European Association of Cardiovascular Imaging (EACVI) [21]. Blood pressure (BP) and heart rate were measured at the end of the exam.

LV quantitative analysis was performed according to guidelines [22]. Relative wall thickness and LV mass were computed by two-dimensional guided M-mode imaging or directly from two-dimensional parasternal long-axis view. Left atrial (LA) volume and LV mass were indexed for body surface area [23]. LV ejection fraction (EF) was computed by measuring LV end-diastolic and end-systolic volumes with the biplane method in apical four- and two-chamber view [22]. For the evaluation of LV diastolic function, Doppler-derived transmitral inflow early (E) and atrial (A) peak velocities (m/s), E/A ratio, E velocity deceleration time (DT), pulsed tissue Doppler of septal and lateral annulus early diastolic velocity (e’), and average E/e’ ratio were determined in apical four-chamber view according to current recommendations [23,24]. LV STE acquisition was obtained in the three apical views (long-axis, four-chamber, and two-chamber) [25,26]. Postprocessing was performed off-line, on a dedicated workstation (EchoPAC only software version 113, GE Healthcare, Horten, Norway). The tracing of endocardial and epicardial borders was defined using automated 2D strain software, with possible manual readjustment when needed. Peak longitudinal strain was measured from six segments in each of the three apical views, and global longitudinal strain (GLS) derived as the average of the individual peak strain before the aortic valve closure. Reproducibility of GLS in our laboratory was previously reported [26,27].

In a non foreshortened apical four-chamber view oriented to obtain the maximal RV internal chamber size, RV diameters (transverse basal and midcavity diameters and longitudinal diameter) were measured at end-diastole. RV systolic function was assessed measuring tricuspid annular systolic excursion (TAPSE, mm) by M-mode echo. Pulsed Doppler RV inflow was recorded to measure tricuspid early diastolic (E) and atrial (A) peak velocities (m/s) and E/A ratio [22]. RV GLS was determined by off-line post-processing by averaging values of the six segments: three of free lateral wall and three of interventricular septal wall [18,28]. Pulmonary arterial systolic pressure (PASP) was estimated according to guidelines, based on the tricuspid regurgitation peak velocity and adding an estimate of right atrial pressure (RAP) by measuring the size and respiratory reactivity of the inferior vena cava (IVC): (a) normal RAP (≈ 5 mmHg) based on a normal IVC size (IVC diameter <2.1 cm) with normal inspiratory collapse (>50% decrease in IVC diameter); (b) RAP ≈ 10 mmHg: dilated IVC (diameter >2.1 cm) or <50% collapse; (c) RAP ≈ 15 mmHg: both dilated IVC and <50% collapse; (d) RAP ≈ 20 mmHg: dilated IVC without visible collapse [23]. The cut-off point of PAH was established for an estimated PAPS >30 mmHg.

Notably, LV and RV GLS values were considered positive (sign +) to strengthen the clinical meaning: the higher the values, the better the strain deformation.

### 2.4. 3D Echocardiography

3D echocardiographic examination of the right ventricle was performed according to previously described procedures [29,30]. A full-volume scan was acquired by harmonic imaging from an apical approach, using a frame rate (in volume per second) higher than 40% of the individual heartbeat or greater than 25 frames per second. Four ECG-gated consecutive heart beats were acquired during an end-expiratory apnea (multibeat acquisition) to generate the full volume. The quality of acquisition was verified in 12-slice display mode in order to exclude stitching artifacts and to ensure the entire RV cavity and walls were included in the full volume and optimal RV border visualization. Adequate data sets were stored digitally in raw data format and exported to EchoPAC only software version 113 (GE Healthcare, Horten, Norway) and elaborated with a commercially available software (4D RV-Function, TomTec Imaging Systems, Gmbh, Unterschleissheim, Germany) for off-line analysis. Every RV full-volume 3D dataset was automatically cropped in three standard planes (views): four-chamber, coronal, and sagittal. Endocardial border was traced at end-diastole and end-systole for the three selected RV planes and served for initiation of automated border detection algorithm. RV contours were automatically traced over the entire cardiac cycle providing quantification of RV end-diastolic volume, RV end-systolic volumes (RV EDV and ESV, respectively) and RV EF. Three-dimensional measurements of RV function have been demonstrated to be highly reproducible in our laboratory [30] and have been also validated against cardiac MRI [31].

### 2.5. Statistical Analysis

Statistical analysis was performed by SPSS package, release 12 (SPSS Inc., Chicago, IL, USA). Data are presented as mean value ± SD. Intergroup comparisons were performed using ANOVA and Bonferroni post-hoc test. Pearson’s correlation was used to evaluate univariate correlates of a given variable. Multivariable linear regression analyses were performed to examine the independent correlates between DLCO_sb_ values and RV after adjusting for confounders such as age, LVEF, and PASP. The null hypothesis was rejected at *p* ≤ 0.05.

## 3. Results

Since the data are normally distributed, they are presented as mean with standard deviation. Demographics, clinical features, and main baseline heart and lung function parameters of the study population are summarized in Table 1. All ILDs patients had a mild-to-moderate lung function deterioration and were able to perform correctly the maneuvers requested for lung assessment. None of them was using high-flow oxygen supplementation. They were also free of cardiac symptoms/signs. Patients and healthy controls had similar age, while body mass index, heart rate, and systolic BP were significantly higher in IPF patients than in controls. Lung function parameters, including 6-MWT, did not differ between IPF and no-IPF patients. History of smoking habits was present in 25 patients (75%) with IPF and 22 (78%) in the no-IPF population. In the IPF population, 16 (48%) had arterial systemic hypertension and 4 (12%) had type II diabetes. In the no-IPF population, 12 patients (43%) had arterial systemic hypertension and one patient (3%) had type II diabetes. Gastroesophageal reflux disease was present in 10 patients with IPF (30%) and in 5 (18%) no-IPF patients.

LV standard echocardiographic and STE assessment are reported in Table 2. LV mass index, relative wall thickness, and LA volume index were comparable between the three groups. Transmitral E/A ratio and septal and lateral e’ velocity were significantly lower in IPF and no-IPF compared to controls. E/e’ ratio was higher in the IPF group (*p* = 0.004), but not in the no-IPF group, compared to controls. LV EF did not differ significantly between IPF, no-IPF, and controls, whereas LV GLS was lower in IPF than in no-IPF (*p* = 0.003) and controls (*p* < 0.0001).

The prevalence of PAH was 65% (*n* = 21) in the IPF group and 75% (*n* = 21) in the no-IPF group but significative PAH (i.e., PASP>50 mmHg) was observed only in four patients (12.5%) with IPF, and in two patients (7%) with no-IPF. RV standard, STE, and 3D analysis are summarized in Table 3. Compared to healthy controls, IPF patients had larger RV transverse (*p* = 0.028) and midcavity (*p* = 0.051) diameters and lower TAPSE (*p* = 0.007). Both IPF and no-IPF patients had lower tricuspid inflow E/A ratio (*p* < 0.001 and *p* < 0.002, respectively) and higher PASP (*p* < 0.002 and 0.047, respectively) versus controls. Both IPF and no-IPF patients had also lower RV GLS in comparison with controls (*p* < 0.001 and *p* < 0.05, respectively) but RV GLS was lower in IPF than in no-IPF (*p* < 0.05). Three-dimensional-derived RV EF was lower in IPF and no-IPF compared to controls (both *p* < 0.002) but did not differ significantly between IPF and no-IPF (*p* = 0.987). 

**Figure 1:**
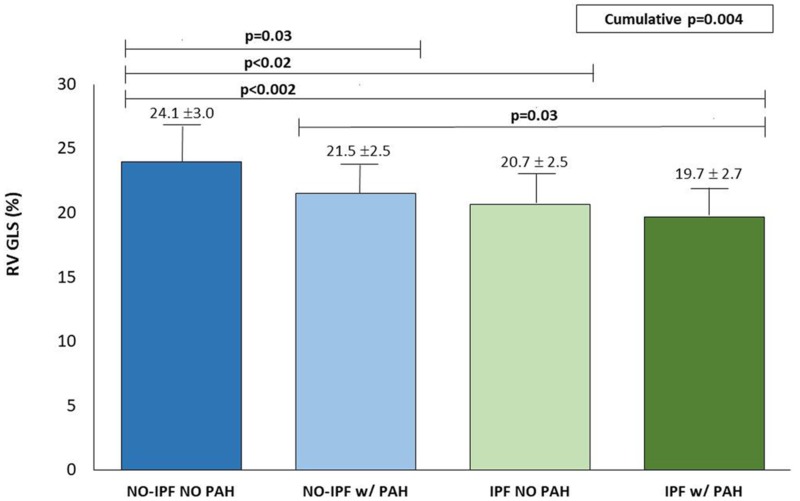
Behavior of RV GLS (mean ± SD) in no-IPF and IPF without and with PAH. RV GLS is significantly lower in IPF with or without PAH in comparison with both no-IPF groups.

Figure 1 depicts subgroup analysis showing the trend of RV GLS in IPF and no-IPF patients with and without PAH. Noteworthy, RV GLS was gradually reduced passing from no-IPF without and with PAH to IPF patients but did not differ significantly between IPF without and with PAH.

### Univariate and Multivariate Associations

**Figure 2:**
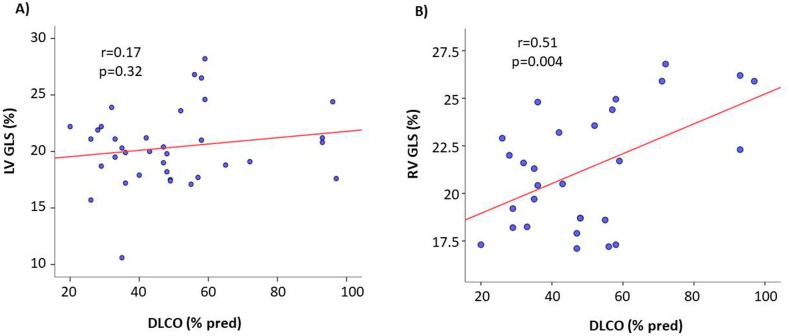
Scatterplot and regression line of the relation between DLCO and both LV GLS and RV GLS in the pooled ILDs population. The relation of RV GLS—but not of LV GLS—is significant.

In the pooled ILDs population, DLCO_sb_ was significantly related with RV GLS (*r* = 0.51, *p* = 0.004), whereas the relation between LV GLS and DLCO_sb_ was not significant (*r* = 0.17, *p* = 0.32) (Figure 2). RV GLS was also negatively related with PASP (*r* = −0.25, *p* = 0.02). The relations of RV GLS with pO_2_ (*r* = 0.03, *p* = 0.83), pCO_2_ (*r* = −0.26, *p* = 0.08), FVC (*r* = 0.04, *p* = 0.79), and TLC (*r* = −0.01, *p* = 0.96) did not achieve statistical significance. Of note, RV EF was not significantly related with any of the spirometric and pulmonary functional parameters. 6-MWT did not correlate with any of the echocardiographic parameters.

By a multivariable regression performed in the pooled ILDs population, after adjusting for body mass index, heart rate, and PASP, DLCO_sb_ was independently associated with RV GLS (standardized β coefficient = 0.583, *p* < 0.0001). These results were substantially confirmed in the IPF subgroup (β = 0.708, *p* < 0.001) whilst no independent association between RV GLS and DLCO_sb_ was found in the no-IPF population (Table 4).

## 4. Discussion

To the best of our knowledge, the present study is the first to compare LV and RV echocardiographic features of IPF and no-IPF patients without evidence of any other heart disease, in relation with a healthy control group. Our findings demonstrate that IPF presents (I) LV diastolic dysfunction, which is detectable even in no-IPF patients, and a subclinical LV systolic dysfunction, testified by the reduction of LV GLS but not of LV EF, which cannot be observed in no-IPF patients; (II) a clear alteration of RV geometry and of both systolic (RV EF and GLS) and diastolic function, and a substantial PASP increase in comparison with controls, whereas no-IPF patients present only an alteration of RV diastolic dysfunction and a lower degree of PASP increase. Moreover, (III) an independent association between DLCO_sb_ and RV longitudinal dysfunction is found in the pooled ILDs population, it being evident in the IPF group but not the no-IPF group.

### 4.1. LV Diastolic and Systolic Function

LV diastolic dysfunction, characterized by a reduction of transmitral E/A ratio and e’ mitral annular velocity and increase of E/e’ ratio, was already observed in IPF patients [9]. Our data confirm this impairment of LV filling (lower E/A ratio) and of myocardial relaxation (lower tissue Doppler septal and lateral e’ velocity) and the higher E/e’ ratio in IPF than in the healthy controls. According to the ASE/EACVI recommendations [24], these findings provide evidence of a variable degree of LV diastolic dysfunction and a trend of LV filling pressure increase in IPF patients. To a lower extent, the same diastolic abnormalities were observed also in non-IPF patients, highlighting a likely process of ventricular diastolic interdependence in all ILDs populations. Ventricular interdependence corresponds to the forces transmitted from the left ventricle to the other and vice versa through the myocardium and pericardium and occurs because the two ventricles have common myocardial fibers, share the interventricular septum, and are wrapped within the pericardium [32]. Accordingly, RV diastolic function can influence that of the left ventricle by several mechanisms including RV pressure overload [33,34,35]. However, in the present study, LV diastolic alterations of both IPF and no-IPF patients were evident in the presence and in the absence of PAH and cannot be therefore ascribed to an impairment of LV myocardial diastolic properties occurring as the consequence of septal wall distortion towards the left ventricle due to RV pressure overload [33,36,37]. It is also worthy of note that only in IPF patients, LV GLS, but not LV EF, was significantly lower in comparison with both healthy controls and no-IPF. This finding is confirmatory of a previous report of D’Andrea et al. on IPF [13] and extends the interdependence phenomenon of IPF to LV longitudinal systolic function. It is conceivable that in this clinical setting, LV systolic involvement could be functional rather than related to structural LV changes as LV mass and relative wall thickness did not differ significantly from controls and no-IPF. This concept is indirectly testified by the reversibility of relief of the RV pressure overload occurring in ILDs patients after lung transplantation [38].

### 4.2. RV Size and Function

RV abnormalities have been demonstrated to be evident and predict prognosis in IPF. In a retrospective study on 135 IPF patients referred for lung transplantation, an increase of RV/LV ratio, right atrial and RV dilation, and moderate to severe RV dysfunction (TAPSE <1.6 cm) were all associated with an increased risk of death, independently of lung function parameters [8]. RV abnormalities appear to be mainly due to PAH, which has a high prevalence (up to 85%) in the advanced disease stages [39]. In IPF patients of less advanced stages (with normal TAPSE and LV EF), D’Andrea et al. showed a significant reduction of RV strain, as an index of early impairment of RV systolic function [12]. RV GLS was also a powerful independent determinant of functional capacity during the six-minute walking test and a cut-off value of RV GLS ≤12% emerged as an independent predictor of cardiac outcome at 19 months follow-up, even in the absence of PAH [13]. In the present study, both IPF and no-IPF patients had lower tricuspid inflow E/A ratio, higher PASP, and lower RV GLS compared to controls. However, a significant difference in RV size (increased RV transverse basal and midcavity diameters) and function (reduced TAPSE) was found only in the IPF when compared to controls. Among the different echocardiographic parameters investigated, only RV GLS differentiated the two ILDs subgroups, it being significantly lower in IPF than in no-IPF. Of note, 3D-echocardiographic-derived RV EF had not the same diagnostic ability since it was significantly reduced in both IPF and no-IPF in comparison with healthy controls. Combined, these findings are completely new and provide evidence of the additional diagnostic capabilities of RV strain imaging in ILDs. It is conceivable that microvascular injury, largely demonstrated as an early stage of IPF lungs [40], could be extended also to the RV subendocardial layer, of which GLS is a reliable marker [41].

Univariate and multivariate associations provided additional insights. The lack of association between FVC and RV GLS was partially unexpected as FVC is more feasible and reproducible than DLCO and largely adopted in the clinical practice and in clinical trials as a predictor of mortality in ILDS [2]. Conversely, the association between DLCO_sb_ reduction and RV GLS impairment, found in the pooled ILDs and in IPF but not in no-IPF, was independent of confounders such as body mass index, heart rate, and PASP. DLCO_sb_ is more strictly associated with pulmonary fibrotic changes and represents, therefore, the best index of the IPF disease status and extension [42] and represents an independent predictor of mortality in patients with ILDs, it being used to stratify the risk in this clinical setting [8,42,43]. It is conceivable, therefore, that the impairment of RV longitudinal function in IPF patients could be essentially due to a reduced gas exchange between alveoli and capillary blood stream.

### 4.3. Study Limitations

First, despite being realized in a highly specialized reference setting, the present study is representative of the effort of a single center with a relatively small sample size of ILDs. As we mainly focused on IPF and smoke-related no-IPF pneumonias, our results certainly do not allow driving definite considerations in other forms of no-IPF including those related to autoimmune, connective tissue diseases. Indeed, patients with connective tissue disease-derived fibrotic ILDs likely merit separate analysis because of their high frequency of concomitant pulmonary vascular involvement [44,45]. In addition, our findings cannot be extrapolated to patients with the most advanced lung fibrosis, as most of our patients were affected by mild to moderate disease.

Another limitation could correspond to the use of DLCO_sb_. Unlike FVC, DLCO changes are not specific as they depend on both ventilation and perfusion and are an expression of the integrity of the alveolar-capillary membrane. In addition, DLCO_sb_ has a marked within-session and intersession variability [13]. Nevertheless, DLCO_sb_ is widely recognized as a surrogate measure of pulmonary fibrosis extent and its changes have been demonstrated to be predictive of IPF disease progression over a 12-month period [3]. Someone could also argue that VC and DLCO_sb_ are not feasible in ILD but this is true in the advanced disease stages and in particular in those requiring long-term oxygen therapy at high oxygen flows, whereas our population included clinically stable ILD patients at the time of first diagnosis.

## 5. Conclusions

The novel findings of the present study point out the diagnostic capabilities of strain imaging in distinguishing early cardiac damage in IPF from no-IPF patients, a task which cannot be attributed to both standard and 3D echocardiography. RV GLS is a key parameter for detecting early cardiac damage in ILDs, with a likely prognostic power, in this clinical scenario. Future studies could be addressed to investigate if RV GLS alterations are also associated with fibrotic heart rearrangement [46].

## Figures and Tables

**Table 1:** Clinical features and baseline heart and lung function parameters of the study population.

Variable	IPF (*n* = 33)	No-IPF (*n* = 28)	*p_a_*	Controls (*n* = 30)	*p_b_*	*p_c_*
Gender (F/M)	6/27	10/18	-	11/19	-	-
Age (years)	70.1 ± 7.6	65.2 ± 8.1	0.067	66.9 ± 8.7	0.375	1.0
BMI (kg/m^2^)	27.8 ± 3.8	30.1 ± 4.0	0.068	25.2 ± 3.2	0.015	<0.0001
Systolic BP (mmHg)	138.8 ± 18.6	133.7 ± 15.2	0.642	128.4 ± 12.5	0.033	0.618
Diastolic BP (mmHg)	78.9 ± 12.2	77.5 ± 9.8	1.0	78.7 ± 9.3	1.0	1.0
Heart rate (bpm)	76.8 ± 11.5	75.7 ± 10.9	1.0	68.2 ± 11.1	0.009	0.039
PaO_2_(mm Hg) in ambient air	71.6 ± 10.1	73.1 ± 8.7	0.548	-	-	-
PaCO_2_(mm Hg) in ambient air	39.7 ± 3.7	38.1 ± 3.0	0.327	-	-	-
FVC (% pred)	71.1 ± 20.9	70.6 ± 19.4	0.227	-	-	-
TLC (% pred)	70.9 ± 47.8	70.1 ± 25.9	0.368	-	-	-
DLCO_sb_ (% pred)	44.3 ± 17.6	54.7 ± 22.3	0.242	-	-	-
6-MWT distance (m)	420.9 ± 168.8	398.5 ± 154.3	0.381	-	-	-

*p_a_* = IPF vs. non-IPF; *p_b_* = IPF vs. controls; *p_c_* = non-IPF vs. controls. IPF = idiopathic pulmonary fibrosis; BMI = body mass index; BP = blood pressure; PaO_2_ = arterial oxygen partial pressure; PaCO_2_ = arterial carbon dioxide partial pressure; FVC % = forced vital capacity; FEV1% = forced expiratory volume in 1s; TLC = total lung capacity; DLCO_sb_ = single-breath lung diffusion capacity of carbon monoxide; 6-MWT: six-minute walking test.

**Table 2:** Standard and advanced echocardiographic parameters of the left ventricle.

Variable	IPF (*n* = 33)	No-IPF (*n* = 28)	*p_a_*	Controls (*n* = 30)	*p_b_*	*p_c_*
LV mass index (g/m^2^)	89.6 ± 19.6	79.9 ± 20.9	0.179	77.8 ± 21.3	0.121	0.938
Relative wall thickness	0.38 ± 0.05	0.38 ± 0.06	0.996	0.36 ± 0.06	0.605	0.568
LV EF (%)	61.1 ± 4.9	63.5 ± 4.9	0.230	62.5 ± 4.9	0.624	0.786
LV GLS (%)	19.5 ± 3.2	22.02 ± 2.4	0.003	22.7 ± 2.6	<0.0001	0.606
Transmitral E/A ratio	0.71 ± 0.15	0.79 ± 0.20	0.314	0.97 ± 0.25	<0.0001	<0.005
E velocity DT (m/s)	258.2 ± 60.6	238.1 ± 47.6	0.414	234.5 ± 74.9	0.310	0.975
Septal e′ velocity (cm/s)	6.0 ± 2.0	7.0 ± 2.0	0.241	9.0 ± 3.0	<0.0001	0.003
Lateral e′ velocity (cm/s)	8.0 ± 2.0	8.0 ± 2.0	0.563	11.0 ± 3.0	<0.0001	<0.01
E/e’ ratio	10.2 ± 4.4	8.7 ± 1.9	0.146	7.5 ± 2.0	0.004	0.336
LAVi (mL/m^2^)	25.5 ± 7.6	23.8 ± 5.5	0.608	25.7 ± 6.4	0.985	0.539

*p_a_* = IPF vs. no-IPF; *p_b_* = IPF vs. controls; *p_c_* = no-IPF vs. controls; DT = deceleration time; LAVi = left atrial volume index; LVEF = left ventricular ejection fraction; LV GLS = left ventricular global longitudinal strain. Other abbreviations as in Table 1.

**Table 3:** Standard and advanced echo-Doppler parameters of the right ventricle.

Variable	IPF (*n* = 33)	No-IPF (*n* = 28)	*p_a_*	Controls (*n* = 30)	*p_b_*	*p_c_*
**Standard Echo-Doppler**						
RV basal tract diameter (mm)	39.8 ± 4.5	38.3 ± 5.7	0.574	35.9 ± 7.0	0.028	0.278
RV mid track diameter (mm)	32.6 ± 5.9	30.7 ± 5.9	0.387	29.3 ± 4.6	0.051	0.579
RV longitudinal diameter (mm)	64.2 ± 7.7	61.4 ± 7.6	0.391	61.5 ± 8.7	0.409	0.998
TAPSE (mm)	20.9 ± 2.9	22.4 ± 3.4	0.165	23.4 ± 3.2	0.007	0.453
Tricuspid E/A ratio	0.88 ± 0.36	0.91 ± 0.21	0.958	1.20 ± 0.30	<0.001	<0.002
PASP (mmHg)	39.6 ± 19.8	37.2 ± 8.1	0.514	26.7 ± 4.6	0.002	0.047
**STE and 3D Echocardiography**						
RV GLS (%)	20.0 ± 2.6	22.1 ± 2.8	<0.05	24.2 ± 4.4	<0.001	<0.05
3D RV EDV (mL)	85.5 ± 2.0	81.3 ± 34.7	0.911	78.6 ± 36.0	0.779	0.959
3D RV ESV (mL)	40.6 ± 15.9	38.8 ± 16.6	0.928	33.3 ± 18.3	0.320	0.495
3D SV (mL)	44.9 ± 18.5	42.6 ± 20.6	1.0	45.4 ± 19.0	1.0	1.0
3D RV EF (%)	50.5 ± 9.9	50.9 ± 7.6	0.987	59.1 ± 6.9	<0.002	<0.002

*p_a_* = IPF vs. no-IPF; *p_b_* = IPF vs. controls; *p_c_* = no-IPF vs. controls; RV = right ventricular; TAPSE = tricuspid annular plane systolic excursion; PASP = pulmonary arterial systolic pressure; GLS = global longitudinal strain; EDV = end-diastolic volume; EF = ejection fraction; ESV = end-systolic volume. Other abbreviations as in Table 1.

**Table 4:** Independent determinants of RV GLS by multiple regression analyses.

	In the Pooled ILDs Population ^a^			In IPF Subgroup ^b^		In No-IPF Subgroup ^c^	
Dependent Variable	Covariate	B Coefficient	*p*	B Coefficient	*p*	B Coefficient	*p*
RV GLS	BMI	−0.186	0.214	−0.267	0.139	−0.227	0.339
	HR	−0.027	0.851	−0.045	0.794	−0.190	0.536
	PASP	−0.053	0.712	−0.203	0.239	−0.304	0.277
	DLCO_sb_	0.583	<0.0001	0.708	<0.001	0.219	0.464

(a) Cumulative R^2^ = 0.575, SEE = 2.22%, *p* < 0.007; (b) Cumulative R^2^ = 0.729, SEE = 1.91 %, *p* = 0.009; (c) Cumulative R^2^ = 0.729, SEE = 1.91 %, *p* = 0.009; ILDs = interstitial lung diseases; IPF = idiopathic pulmonary fibrosis; DLCOsb= single-breath lung diffusion capacity of carbon monoxide; PASP = pulmonary arterial systolic pressure; RV = right ventricular; GLS = Global longitudinal strain; SEE = standard error estimated.
